# PCDD/Fs, DL-PCBs, and NDL-PCBs in Dairy Cows: Carryover in Milk from a Controlled Feeding Study

**DOI:** 10.1021/acs.jafc.9b08180

**Published:** 2020-02-05

**Authors:** Valentina Lorenzi, Barbara Angelone, Enrica Ferretti, Andrea Galli, Mauro Tonoli, Matteo Donati, Francesca Fusi, Giorgio Zanardi, Sergio Ghidini, Luigi Bertocchi

**Affiliations:** † Istituto Zooprofilattico Sperimentale della Lombardia e dell’Emilia Romagna “Bruno Ubertini”, Via Antonio Bianchi 9, 25124 Brescia, Italy; ‡ Research Centre for Animal Production and Aquaculture, CREA, Via Antonio Lombardo 11, 26900 Lodi, Italy; § Department of Food Science, Parma University, Via del Taglio 10, 43126 Parma, Italy

**Keywords:** non-ortho DL-PCBs, mono-ortho DL-PCBs, NDL-PCB indicators, feed, cow milk

## Abstract

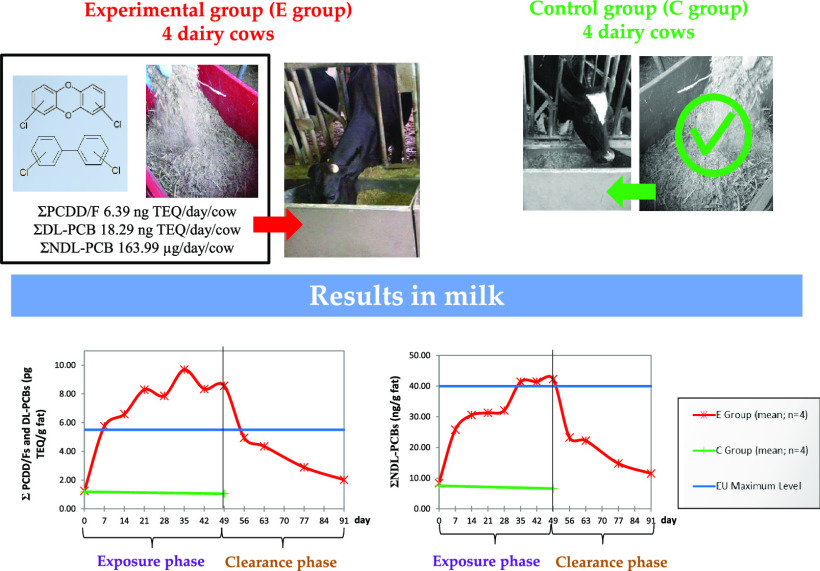

A feeding study was carried out to investigate the kinetics in cow milk of the 17 polychlorinated dibenzo-*p*-dioxins and dibenzofurans (PCDD/Fs), the 12 dioxin-like polychlorinated biphenyls (DL-PCBs), and the 6 non-dioxin-like PCBs (NDL-PCBs) regulated by the European (EU) legislation. A fortified ration (ΣPCDD/Fs and DL-PCBs: 24.68 ng TEQ/day/cow; ΣNDL-PCBs: 163.99 μg/day/cow) was given to the animals for 49 days, followed by 42 days on clean feed. EU maximum limit for TEQ_PCDD/F+DL-PCB_ was exceeded in milk after 1 week of exposure, while for ΣNDL-PCBs, after 5 weeks. Milk compliance was restored after 1 week on clean feed, but to return to the basal TEQ_PCDD/F+DL-PCB_ it took 42 days. At the end of the study, ΣNDL-PCBs had not yet reached the basal level. The carryover rate of ΣNDL-PCBs was 25.4%, while the carryover rate of TEQ_PCDD/F+DL-PCB_ was 36.9%. The latter was mainly affected by the 12 congeners contributing most to the toxic equivalent (TEQ) level, explaining the fast overcome of the maximum limit in milk.

## Introduction

In Europe (EU), environmental and food contaminations by persistent organic pollutants, such as polychlorinated dibenzo-*p*-dioxins and dibenzofurans (PCDD/Fs) and polychlorinated biphenyls (PCBs), are slowly declining thanks to the entry into force of the Stockholm Convention and to the adoption of an EU strategy aimed at decreasing human exposure toward the food chain.^1,2^ Despite the progressive improvement of risk management measures, accidental feed and food contaminations can still happen, due, for example, to the use of contaminated soil for agricultural activities, to the presence of PCBs in open applications, and to the open-air burning of waste.^2−5^ In addition, part of the EU population still exceeds the tolerable weekly intake (TWI) of 14 pg toxic equivalents (TEQ)/kg of body weight (bw), set for the sum of PCDD/Fs and dioxin-like PCBs (DL-PCBs).^2,4,5^ This TWI has been recently revised by the European Food Safety Authority (EFSA) and reduced to 2 pg TEQ/kg bw per week, implying a substantial exceeding of the limit by the European consumers.^6^

The ingestion of food and feed are the primary sources of human and animal exposure to PCDD/Fs and PCBs;^2^ thus, maximum (ML) and action (AL) levels have been set by EU to protect public health.^7−10^ MLs were laid down following the “strict but feasible” principle; thus, feed MLs do not take into account the carryover from feed to food.^2^ As a consequence, feed slightly below EU MLs could result in food contamination above MLs, as already demonstrated for eggs and beef meat.^2−4,11^

Among food of animal origin, milk and dairy products remain an important source of PCDD/F and PCB exposure for humans, because of their high consumption rate.^12,13^ In particular, cow milk has been often involved in several cases of pollution, e.g.,^10,14−16^ demonstrating the vulnerability of the milk chain to these contaminants.^12^

Given the important role of cow milk in human exposure, several carryover experiments in dairy cows have been carried out. However, there are very few papers reporting recent controlled feeding studies in dairy cows and describing both an exposure and an elimination phase.^6,12^ Most of the studies date back to the last century, e.g.,^17−21^ and, to our knowledge, no one dealt with all 35 PCDD/F and PCB congeners, currently regulated by the European legislation.^7−9^

The present paper describes a controlled feeding study involving a group of lactating cows, which were fed a total mixed ration (TMR), fortified with a known amount of the 17 2,3,7,8-substitued PCDD/Fs, the 12 DL-PCB congeners (PCB 77, 81, 105, 114, 118, 123, 126, 156, 157, 167, 169, and 189), and the 6 non-dioxin-like PCB indicators (NDL-PCBs: PCB 28, 52, 101, 138, 153, and 180). The fortified ration was given to the animals (exposure phase) until the milk exceeded the EU MLs for the sum of PCDD/Fs and DL-PCBs and for NDL-PCBs;^7^ then, the depletion of the contaminants was monitored (clearance phase). A pharmacokinetic approach was used to analyze the excretion in dairy cow milk of PCDD/Fs, DL-PCBs, and NDL-PCBs: steady state (SS), carryover rate (COR), and the time needed to return to the basal level were determined for the 35 investigated congeners. The final goal was to acquire additional data for improving the management of cow milk contamination in areas with a history of pollution or during accidental episodes.

## Materials and Methods

The experimental protocol was approved by the Ethics Committee of the Istituto Zooprofilattico Sperimentale della Lombardia e dell’Emilia Romagna, during the session of 12 December 2013 (request code 14-4-13), and the approval was transmitted to the Italian Ministry of Health (protocol number 5166_2014).

### Chemicals

Native and ^13^C-labeled standards were purchased from Cambridge Isotope Laboratories (Tewksbury, Massachusetts) and Dr. Ehrenstorfer (Augsburg, Germany). Prepacked multilayer silica, alumina, and carbon columns were produced by Fluid Management System (Lexington, Kentucky). Ethyl-acetate, toluene, and nonane were purchased from Promochem (LGC Standards, Teddington, U.K.); dichloromethane from ROMIL (Waterbeach, U.K.), and *n*-hexane from J.T.Baker (Avantor Performance Materials, Radnor Township, Pennsylvania). All solvents were picograde.

### Selection of the Animals

The feeding study was carried out in 2014 in the teaching farm of an agricultural high school located in Brescia (North Italy). This city was the site of the former Italian PCB-producing plant, which polluted the soil and the irrigation ditches of an area of the town, later recognized as National Priority Contaminated Site.^22^ PCB contamination was found in the locally produced food, including cow milk.^16,23^

The loose-housing farm, involved in the study, had around 60 lactating cows (Italian Holstein-Friesian breed). Before starting the study, the levels of contamination for PCDD/Fs, DL-PCBs, and NDL-PCBs of the bulk tank milk were tested to verify milk background levels. Eight healthy lactating cows belonging to the herd were recruited for the experiment. Selection of the cows was based on the date of birth, date of calving, number of lactations, and milk yield, to create 2 homogeneous groups of 4 cows each: the experimental group (E) and the control group (C). The characteristics of the selected cows are reported in Supporting Information Table S1. Each group included 2 cows in the first 100 days of lactation (one primiparous cow and one secondiparous cow) and 2 cows over the first 100 days of lactation (one primiparous cow and one secondiparous cow). C group was used to exclude the contribution of other possible sources of contamination (e.g., air, water, straw bedding, etc.) to the total exposure, during the whole experiment.

The two groups were housed separately from the main herd, in two adjacent pens of the same size. Each pen was characterized by 4 feeding places (self-catching feed rack) with 4 steel mangers, one drinking bowl, straw bedding, and access to an outside loafing area with concrete floor. It was not possible for each group of cows to take feed from the other group or from the other cows within the farm.

Ten days before the starting of the experiment, the cows were moved into the pens to get used to the new environment and to the new ration.^12,17,24^ Both groups of cows were fed with a TMR purchased from a specialized supplier (Consorzio Agrario Cremona, Cremona, Italy) to guarantee homogenous feed composition. To avoid changing in the raw material, the entire amount of TMR, needed to carry out the feeding study, was bought and stored in the farm facilities, according to good agricultural practices. Before starting the administration to the animals, TMR was analyzed to determine PCDD/F and PCB background contamination levels. During the conditioning period and throughout the experiment, a total of 23 kg/cow of TMR at 88% dry matter (DM) was given daily to the animals after the morning milking. Cows were milked twice a day (6.00 am/6.00 pm) in a double-4 parlor (DeLaval, Tumba, Sweden). During the whole study, E group was always milked at the same milking stall lane, while C group occupied the opposite lane. The milking machine was automatically and accurately washed before and after each milking session, using hot water and a chlorine free alkaline detergent, alternated with an acid detergent, to remove milk residues.

Once a week, the animals were weighed to monitor possible changes in the body weight that might have influenced body fat storage and, as a consequence, the excretion of the contaminants in milk.^18−21^

### Source of PCDD/F and PCB Contamination

To study the uptake and the excretion of PCDD/Fs and PCBs in dairy cows under controlled conditions, corn oil was used as a contaminant carrier. This choice was justified by the fact that corn oil is highly palatable to dairy cattle, it is easy to purchase, and it has generally a very low content in PCDD/Fs and PCBs, enough to be suggested as a reference matrix method blank by the Environmental Protection Agency of the United States.^25,26^

Ten liters of corn oil, sold for human nutrition, was purchased for the research study and a sample (500 mL) was tested to confirm the absence of significant levels of contamination. Four and a half liters were aliquoted (20 mL aliquots) and stored at room temperature (blank corn oil). Other five liters were artificially contaminated in laboratory by adding a known amount of native standards. Standard mixtures containing the 17 PCDD/Fs (EDF-7999-10×, Cambridge Isotope Laboratories) and the 12 DL-PCBs (PCB-Mix 41, Dr. Ehrenstorfer) and native standards of the 6 NDL-PCBs (PCB No. 28; PCB No. 52; PCB No. 101; PCB No. 153; PCB No. 138; PCB No. 180, Dr. Ehrenstorfer) were dissolved in corn oil. Then, the artificially contaminated corn oil was aliquoted (20 mL) to be administered during the experimental protocol.

The concentration of the contaminants added to the corn oil is reported in Table 1. During the selection of the contaminant dose, priority was given to DL-PCBs rather than to PCDD/Fs; in fact, it was chosen to skew the contamination toward DL-PCBs to better understand their kinetics and their carryover from feed to cow milk, since the literature on this field was rather scarce for PCBs. In particular, it was chosen to administrate to the animals a dose with a “TEQ ratio” (defined as the relation between DL-PCB TEQ divided by PCDD/F TEQ)^27^ of about 3 to 1, to resemble the average TEQ ratio found in the local forages.^28^ In fact, Turrio-Baldassarri et al.^28^ reported a PCDD/F mean contribution to the total TEQ (sum of PCDD/Fs and DL-PCBs) of 27.8% (range 14.3–43%) in the forages collected in the agricultural area of Brescia around the PCB-producing plant.

**Table 1:** PCDD/Fs, DL-PCBs, and NDL-PCBs Dissolved in the Artificially Contaminated Corn Oil

concentration in corn oil (ng/g)			
PCDD/Fs		PCBs	
**PCDFs**		**DL-PCBs**	
2,3,7,8-TCDF	0.03	PCB 81	7.61
1,2,3,7,8-PeCDF	0.15	PCB 77	7.61
2,3,4,7,8-PeCDF	0.15	PCB 123	7.61
1,2,3,4,7,8-HxCDF	0.15	PCB 118	7.61
1,2,3,6,7,8-HxCDF	0.15	PCB 114	7.61
2,3,4,6,7,8-HxCDF	0.15	PCB 105	7.61
1,2,3,7,8,9-HxCDF	0.15	PCB 126	7.61
1,2,3,4,6,7,8-HpCDF	0.15	PCB 167	7.61
1,2,3,4,7,8,9-HpCDF	0.15	PCB 156	7.61
1,2,3,4,6,7,8,9-OCDF	0.30	PCB 157	7.61
**PCDDs**		PCB 169	7.61
2,3,7,8-TCDD	0.03	PCB 189	7.61
1,2,3,7,8-PeCDD	0.15	**NDL-PCBs**	
1,2,3,4,7,8-HxCDD	0.15	PCB 28	1669.57
1,2,3,6,7,8-HxCDD	0.15	PCB 52	1643.91
1,2,3,7,8,9-HxCDD	0.15	PCB 101	1549.57
1,2,3,4,6,7,8-HpCDD	0.15	PCB 153	1670.26
1,2,3,4,6,7,8,9-OCDD	0.30	PCB 138	735.43
		PCB 180	1643.91

The employed standard mixtures determined the congener patterns of PCDD/Fs and DL-PCBs in the fortified corn oil. In particular, the DL-PCB mixture allowed to have the same contribution of each congener to the total concentration of DL-PCBs, to better understand how the initial feed pattern of contamination would have resulted in cow milk.

No local data were available for NDL-PCB contamination of feed; thus, the NDL-PCB pattern was chosen on the basis of both the availability of the native standards and on the data published by EFSA in compound feed, which reported an equal contribution of the single NDL-PCBs to the total concentration.^29^

For the selection of the contamination level, corn oil was considered as a feed included in the TMR; thus, starting from the amount of feed daily offered to the cows (23 kg of TMR at 88% DM + 20 mL of corn oil), a contamination level was selected that guaranteed to have a TMR under the MLs set by the European Commission in compound feed,^8^ to reproduce a legislative compliant TMR that could potentially be used on farm or sold in the market.

### Experimental Design

After the 10 days of conditioning period, C group was fed daily with the purchased TMR (23 kg/cow) and 20 mL/cow of blank corn oil while E group was fed with the same TMR (23 kg/cow) but fortified with 20 mL/cow of artificially contaminated corn oil. The administration of the fortified ration to E group began on 7 January 2014, after the morning milking. Cows’ days in milk on that date (Time zero—*T*_0_) are reported in Supporting Information Table S1. Before starting the exposure phase, the milk of the 8 cows was sampled and analyzed to define the background contamination levels at *T*_0_. For each cow, a sample of milk was obtained by combining 500 mL of milk from the evening milking of January 6 and 500 mL of milk from the morning milking of January 7.

During the exposure phase, every day, after the morning milking, 20 mL of blank corn oil was mixed with 1 kg of TMR and supplied to each cow of C group, locked in the feed barrier, using one plastic bowl per cow positioned in the corresponding steel manger. The same was done with each cow of E group but using 20 mL of artificially contaminated corn oil. Once all cows finished their ration, the plastic bowls were removed from the mangers, the cows were unlocked from the feed barrier, and 22 kg/cow of TMR was supplied to each group. These operations were carried out every day simultaneously by two different operators to avoid possible cross contaminations. The fortified ration was given until the milk of E group exceeded the MLs for the sum of PCDD/Fs and DL-PCBs and for NDL-PCBs. After that, both groups were fed with the not fortified TMR and the E group clearance phase was studied until the milk contamination decreased under the ALs.

It was not possible to measure the individual feed intake, but the daily average feed ingestion rate of each group of animals was monitored by weighing the feed offered to the group (23 kg/cow per day) and the feed remaining the day after.

### Collection of the Samples

TMR was sampled once, at the beginning of the conditioning period, following the provisions of Commission Regulation (EU) No 152/2009,^30^ modified by Commission Regulation (EU) No 691/2013.^31^ The sample was immediately delivered to the laboratory for the analysis.

Cow milk was collected according to Commission Regulation (EU) No 252/2012.^32^ The milk of each cow was sampled at *T*_0_ and then once a week, during the exposure phase. At the clearance phase, milk was sampled once a week during the first 2 weeks of depletion and then every 2 weeks. The milking machine (DeLaval, Tumba, Sweden) allowed the collection of homogeneous samples of milk from each cow and the measurement of the individual milk yield. At each milking sampling point, separate milk samples from each cow were collected during the evening milking (500 mL/cow) and during the following morning milking (500 mL/cow). Samples were stored at −20 °C until analysis. Milk yields were recorded at each milk sampling point. Unsampled milk was disposed as category 1 material, according to Regulation (EC) 1069/2009.^33^

### Analysis of the Samples

For the quantitative determination of PCDD/Fs and PCBs, samples of TMR, blank corn oil, and milk were analyzed using high-resolution gas-chromatography coupled with high-resolution mass spectrometry (HRGC-HRMS) (Thermo Fisher Scientific, Waltham, Massachusetts). The laboratory performing the analysis was certified under UNI CEI ISO/IEC 17025 and accredited for the determination of the 35 investigated molecules. Analysis of the samples was performed as described in Lorenzi et al.^22^

Briefly, the TMR sample (5 g) and the blank corn oil sample (3 g) were mixed with diatomaceous earth, spiked with a mixture of 15 ^13^C-labeled PCDD/Fs and 12 ^13^C-labeled PCB congeners and then extracted with an Accelerated Solvent Extractor (ASE) (Dionex, Sunnyvale, California), using toluene.

Milk samples of each cow were prepared for the analysis by mixing the aliquot obtained from the evening milking (500 mL) with a same amount collected during the following morning milking. The obtained samples were homogenized, freeze-dried (Freeze Dryer Martin Christ, Osterode am Harz, Germany), and homogenized again. Milk from E group cows was processed separately from C group one to avoid cross contamination. A portion of the milk samples underwent Soxhlet method for the determination of the lipid content, while 8–10 g of powder was mixed with diatomaceous earth and spiked with the ^13^C-isotope labeled standards. Fat was extracted with toluene by means of two cycles at 135 °C and 1500 PSI using ASE.

For all samples, the obtained solvent was filtered through anhydrous sodium sulfate and evaporated with a rotatory evaporator at 45 °C. After overnight drying in oven at 70 °C, the extracts were solubilized with 5 mL of hexane/dichloromethane solution (1:1, v/v), spiked with a clean-up standard solution containing three ^13^C-labeled PCB congeners, and diluted with 20 mL of hexane. Then, the dilute extracts were subjected to a double purification step: (i) the extracts were loaded onto silica columns, acidified with sulfuric acid, and eluted with *n*-hexane; (ii) after evaporation of the hexane, the purification fractions, concentrated to 0.5 mL, were loaded into the Power-Prep system (Fluid Management System, Lexington, Kentucky) equipped with silica, alumina, and carbon columns. Toluene was used to elute PCDD/Fs from the carbon column, while *n*-hexane and a mixture of hexane/dichloromethane solution (9:1, v/v) were used for PCB elution from the alumina column. Each final extract was evaporated to dryness using a TurboVap evaporator (Zymark Corp., Mountain View, California) and a vacuum concentrator (Genevac, Ipswich, U.K.).

The PCDD/F fraction was dissolved in 10 μL of ^13^C-labeled 1,2,3,4-TCDD and ^13^C-labeled 1,2,3,7,8,9-HxCDD injection solution and the PCB fraction, in 20 μL of ^13^C-labeled PCB 52, ^13^C-labeled PCB 101, ^13^C-labeled PCB 138, and ^13^C-labeled PCB 194 injection solution. HRGC-HRMS analysis and quality control were carried out as described by Lorenzi et al.^22^

For TMR and corn oil (i.e., feed), results were expressed in ng/kg with a moisture content of 12% for PCDD/Fs and DL-PCBs and in μg/kg with a moisture content of 12% for NDL-PCBs. For milk, results were expressed in pg/g fat for PCDD/Fs and DL-PCBs and in ng/g fat for NDL-PCBs. Toxic equivalent values (TEQ) were calculated using the World Health Organization-Toxic Equivalency Factors (WHO-TEFs) set in 2005.^34^

### Statistical Analysis

The kinetics of the 35 investigated compounds was studied in E group cows following the model described by Costera and colleagues.^35^ The time needed to reach the SS was determined using the following equation
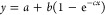
1where *y* is the congener concentration at a given time (pg/g milk fat), *a* is the initial congener concentration (pg/g milk fat), *a* + *b* is the congener concentration at plateau (pg/g milk fat), *c* is the time constant rate, and *x* is the time to reach the SS (days).^35^

COR (%) was calculated as follows
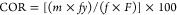
2where *m* is the congener concentration in milk fat at SS (pg/g), *fy* is the fat yield (g/day), *f* is the congener concentration in the feed (pg/g), and *F* is the feed daily intake (g/day).^35^ COR is a useful descriptor of the transfer of PCDD/Fs and PCBs from feed to milk: it includes both feed inputs and food outputs, and it is not strongly influenced by the characteristics of the individual animal.^20,21,36^

In addition, the clearance of PCDD/Fs and PCBs from E group milk was studied using a linear model (LM) to identify the time needed by the compounds to return to the basal level. The following model was used
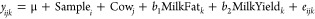
3where *y* is the congener concentration in milk, μ is the overall average, *Sample* is the fixed effect of the *i*th milk sample, *Cow* is the fixed effect of the *j*th cow; *MilkFat* is the effect of the covariate milk fat, *MilkYield* is the effect of the covariate milk yield, and *e* is the residual standard error.

Statistical analysis was performed using R version 3.01.^37^

## Results and Discussion

### Background Exposure of the Animals Employed in the Study

Since the study was carried out in an area with a history of pollution, the presence of PCDD/Fs, DL-PCBs, and NDL-PCBs in the bulk tank milk of the farm involved in the experiment was preliminarily investigated to define the background contamination levels. Results are reported in Supporting Information Table S2; PCDD/F and PCB values were below EU MLs and ALs, and they were in line with previous reported data for cow milk produced in northern Italy.^22^

Moreover, before starting the experiment, the blank corn oil and the TMR, used for feeding C group and E group cows, were tested to exclude the presence of significant levels of contamination that could interfere with the study. In the blank corn oil, 34 out of the 35 investigated molecules were under the limit of quantifications (LOQs) and only 2,3,7,8-TCDF could be quantified (0.05 ng/kg 12% moisture content). Concerning TMR analysis, 32 molecules resulted under the LOQs and only 2,3,7,8-TCDF (0.16 ng/kg 12% moisture content), 1,2,3,4,6,7,8-HpCDD (0.16 ng/kg 12% moisture content), and 1,2,3,4,6,7,8,9-OCDD (0.35 ng/kg 12% moisture content) could be quantified. The upper-bound (UB) and lower-bound (LB) levels for PCDD/F TEQ, DL-PCB TEQ, and NDL-PCB concentration, found in these matrices, are reported in Supporting Information Table S3.

### Cows’ Ingestion Rate, Milk Production, and Body Weight

The experimental study lasted 91 days: 49 days of the exposure phase, followed by 42 days of the clearance phase. At day 67 (third week of the depletion phase), cow 1E was excluded from the study, due to health problems. During the whole study period, no other health problems were diagnosed and no pharmacological treatments were carried out in the other 7 cows involved in the experiment.

Throughout the study, 23 kg/cow of TMR was daily offered to the animals immediately after the morning milking. The daily average ingestion rates of E group and C group cows are reported in Supporting Information Figure S1. During the exposure phase (from day 1 to 49), E group consumed on average 88.3 kg/day of TMR (22.1 kg/cow per day), while C group consumed on average 86.8 kg/day of TMR (21.7 kg/cow per day). TMR daily average consumption showed initially some variation in both groups, then became stable. During the clearance phase (from day 50 to 91), the E group average ingestion rate was affected by the health problems of cow 1E and it showed more fluctuations than C group. During this phase, E group consumed on average 21.1 kg/cow per day of TMR, while C group consumed on average 22.4 kg/cow per day.

Milk samples were collected at days 0, 7, 14, 21, 28, 35, 42, 49 (end of the exposure phase), 56, 63, 77, and 91 (end of the monitoring of the clearance phase). Cow milk production and body weight, recorded at each milk sampling point, are shown in Supporting Information Figures S2 and S3, respectively. Milk production reflected cows’ lactation phase and it showed an overlapping trend between E group and C group. Cows’ body weight stayed rather constant throughout the study.

### Contaminant Excretion in Milk

During the 49 days of exposure, each cow of E group received, with the fortified corn oil, a PCDD/F daily dose of 6.39 ng TEQ/day, a DL-PCB dose of 18.29 ng TEQ/day, and 163.99 μg/day of NDL-PCBs. Considering these amounts against the average daily feed consumption per cow (22.1 kg/cow of TMR at 88% DM) and taking into account the contribution of TMR and corn oil background contaminations, it can be assumed that each cow of E group ingested 1.23 ng TEQ/kg at 12% moisture content for the sum of PCDD/Fs and DL-PCBs (0.33 ng TEQ/kg PCDD/Fs and 0.90 ng TEQ/kg DL-PCBs, respectively) and 7.61 μg/kg at 12% moisture content for NDL-PCBs. These values were under the MLs set by the European Commission^8^ for the sum of PCDD/Fs and DL-PCBs (ML: 1.50 ng TEQ/kg at 12% moisture content) and for NDL-PCBs (ML: 10 μg/kg at 12% moisture content) in compound feed; however, DL-PCB contamination (0.90 ng TEQ/kg at 12% moisture content) was almost twice the specific EU AL (0.5 ng TEQ/kg at 12% moisture content).

Table 2 shows contaminant levels found in the milk samples collected from E group and C group cows. For C group cows, only *T*_0_ and *T*_49_ milk samples were analyzed, to confirm the absence of other significant sources of contamination that could have affected the experimental results during the exposure phase.

**Table 2:** Upper-Bound Levels of PCDD/Fs (pg TEQ/g Fat), DL-PCBs (pg TEQ/g Fat), Sum of PCDD/Fs and DL-PCBs (pg TEQ/g Fat) and of NDL-PCBs (ng/g Fat) in the Milk of Cows 1E, 2E, 3E, and 4E, Belonging to the Experimental Group (E Group), and in the Milk of the Control Cows (1C, 2C, 3C, and 4C)^a^

		treatment days											
		0	7	14	21	28	35	42	49	56	63	77	91
∑PCDD/Fs (pg TEQ/g fat)	1E	0.25	1.18	1.58	2.05	2.00	1.86	1.81	2.00	1.12	1.45	-	-
	2E	0.20	0.91	1.07	1.83	1.48	1.57	1.47	1.44	0.81	0.63	0.36	0.36
	3E	0.22	1.18	1.46	2.08	1.91	2.49	1.96	2.28	0.97	0.89	0.45	0.25
	4E	0.30	1.15	1.03	1.58	1.63	1.66	1.52	1.70	0.92	0.86	0.61	0.43
	**E group mean**	**0.24**	**1.11**	**1.29**	**1.89**	**1.76**	**1.90**	**1.69**	**1.86**	**0.96**	**0.96**	**0.47**	**0.35**
	1C	0.27							0.20				
	2C	0.29							0.25				
	3C	0.24							0.24				
	4C	0.29							0.49				
	**C group mean**	**0.27**							**0.30**				
∑DL-PCBs (pg TEQ/f fat)	1E	0.74	4.04	5.53	7.01	6.08	7.19	6.80	6.54	4.52	4.43		
	2E	0.60	3.57	3.99	5.72	5.36	5.96	5.26	5.37	3.47	2.33	1.99	1.37
	3E	1.18	5.68	6.56	6.96	7.21	9.89	7.71	8.37	4.24	3.55	2.33	1.63
	4E	1.49	5.27	5.15	5.98	5.81	8.15	6.85	6.59	3.76	3.28	2.93	2.07
	**E group mean**	**1.00**	**4.64**	**5.31**	**6.42**	**6.12**	**7.80**	**6.66**	**6.72**	**4.00**	**3.40**	**2.42**	**1.69**
	1C	0.24							0.43				
	2C	0.83							0.68				
	3C	1.13							0.52				
	4C	1.42							1.39				
	**C group mean**	**0.91**							**0.76**				
∑PCDD/Fs and DL-PCBs (pg TEQ/g fat)	1E	0.99	5.22	7.11	9.06	8.08	9.05	8.61	8.53	5.63	5.88		
	2E	0.80	4.48	5.06	7.55	6.84	7.53	6.73	6.81	4.28	2.96	2.35	1.74
	3E	1.40	6.86	8.02	9.03	9.12	12.38	9.67	10.64	5.21	4.43	2.78	1.83
	4E	1.79	6.43	6.17	7.56	7.44	9.81	8.37	8.29	4.68	4.14	3.54	2.49
	**E group mean**	**1.25**	**5.75**	**6.59**	**8.30**	**7.87**	**9.69**	**8.35**	**8.57**	**4.95**	**4.35**	**2.89**	**2.02**
	1C	0.52							0.63				
	2C	1.12							0.93				
	3C	1.37							0.76				
	4C	1.71							1.89				
	**C group mean**	**1.18**							**1.05**				
∑NDL-PCBs (ng/g fat)	1E	7.16	23.24	33.05	32.54	31.99	38.94	42.07	48.37	28.25	27.87		
	2E	7.46	24.81	24.72	27.88	26.76	35.48	35.09	33.45	18.87	18.05	12.92	9.80
	3E	9.49	28.22	35.70	33.96	37.51	49.76	46.43	49.55	25.86	24.03	16.11	12.33
	4E	9.31	26.89	28.72	30.73	32.00	41.27	42.16	37.83	19.84	18.95	15.28	12.34
	**E group mean**	**8.36**	**25.79**	**30.55**	**31.28**	**32.07**	**41.36**	**41.44**	**42.30**	**23.21**	**22.23**	**14.77**	**11.49**
	1C	6.00							6.00				
	2C	7.20							6.02				
	3C	7.77							6.25				
	4C	9.05							8.03				
	**C group mean**	**7.51**							**6.58**				

a E cows received 20 mL/day of fortified corn oil for 49 days (days 1–49). Then, they returned to the basal ration (days 50–91). Cow 1E was excluded from the study at day 67, due to health problems. E group and control group (C group) mean contaminant values are reported in bold.

During the contaminant administration phase, E group cows showed a rapid increase of the milk TEQ levels both for PCDD/Fs and DL-PCBs and of the concentration of NDL-PCBs, followed by gradual stabilization (Table 2). At *T*_7_, E group milk TEQ levels for PCDD/Fs and for DL-PCBs and the concentration in milk of the 6 NDL-PCB indicators were on average 4.56-fold, 4.63-fold, and 3.09-fold greater than those at *T*_0_, respectively, and they were on average more than 50% of the maximum levels found in milk during the exposure phase. The rapid increase of milk contamination, in the feeding study with continued exposure of dairy cows, had been also reported by other authors using Aroclor 1254^38^ or feed and feed supplement (e.g., maize silage, sugar beet pulp, magnesium oxide supplement) contaminated by PCDD/Fs and DL-PCBs.^12,24^ In our study, the same trend was also seen for the sum of the 6 NDL-PCB indicators.

In both E group and C group, the first calving cows (i.e., 3E, 4E, 3C, and 4C) showed generally higher contaminant values in milk at *T*_0_ than secondiparous cows (i.e., 1E, 2E, 1C, and 2C) both for the sum of PCDD/Fs and DL-PCBs (TEQ value) and for NDL-PCBs. The body burden of the primiparous cows, accumulated during their nonproductive life and eliminated during the first lactation, could explain these findings.^39^ However, during the 49 days of the exposure phase, the difference between the first calving cows and second calving cows was not maintained within the E group, because cows 1E and 3E (i.e., cows > 100 days in milk) reached generally higher PCDD/F TEQ, DL-PCB TEQ, and NDL-PCB values than cows 2E and 4E (i.e., fresh cows), respectively (Table 2). In early lactating cows, milk fat production depends mainly on the mobilization of body fat reserves and this mobilization could explain the lower contaminant level recorded in milk from cows 2E and 4E, as a result of a dilution effect.^21,36^

During the exposure phase, E group milk contamination, averaged for the 4 cows, exceeded the EU ML set for the sum of PCDD/Fs and DL-PCBs (5.5 pg TEQ/g fat)^7^ after only one week of exposure (*T*_7_) and it exceeded the EU ML for NDL-PCBs (40 ng/g fat)^7^ after 5 weeks (*T*_35_). E group milk never reached the ML established for PCDD/Fs (2.5 pg TEQ/g fat),^7^ but the corresponding AL (1.75 pg TEQ/g fat)^9^ was overcome at T_21_. The AL for DL-PCBs (2.00 pg TEQ/g fat)^9^ was exceeded at *T*_7_.

C group didn’t show any significant variation of the initial level of contamination (Table 2).

After 1 week (*T*_56_), from the withdrawing of the contaminated diet, milk average levels of E group dropped under the EU MLs both for the sum of PCDD/Fs and DL-PCBs and for NDL-PCBs. E group mean contaminant levels in milk fell under the AL set for PCDD/Fs and the AL set for DL-PCBs at *T*_56_ and *T*_91_, respectively. As shown in Table 2, levels in milk dropped rapidly during the first week, decreasing on average by 48.52% for PCDD/F TEQ, 40.49% for DL-PCB TEQ, and 45.14% for the 6 NDL-PCBs at *T*_56_ compared with *T*_49_ levels; then, the decline was more gradual. After 14 days from the withdrawing of the contaminated corn oil (T_63_), E group milk levels for all three contaminant categories (PCDD/Fs, DL-PCBs, and NDL-PCBs) were on average around 50% of the levels at the end of the exposure phase (*T*_49_). This trend, characterized by an initial rapid decline of milk levels followed by a slow decline, agrees with previous findings,^6,12,24,38,40^ and in the present study, it was shown also by NDL-PCBs (sum of the 6 indicators). At the end of the monitoring of the clearance phase (*T*_91_), after 42 days on clean feed, TEQ contamination levels of milk (sum of PCDD/Fs and DL-PCBs) were reduced by 74.45% for cow 2E, 82.80% for cow 3E, and 69.96% for cow 4E, compared with the levels found at *T*_49_. At *T*_91_, NDL-PCB concentration (sum of the 6 indicators) was reduced by 70.70% for cow 2E, 75.12% for cow 3E, and 67.38% for cow 4E, compared with the levels found at the end of the exposure phase (*T*_49_). Cow 3E (i.e., the first calving cow with more than 100 days in milk) showed the highest percentage reductions at *T*_91_ compared with cows 2E and 4E, but it was also the cow that reached the highest contaminant levels at *T*_49_ (Table 2).

### Congener Patterns

**Figure 1:**
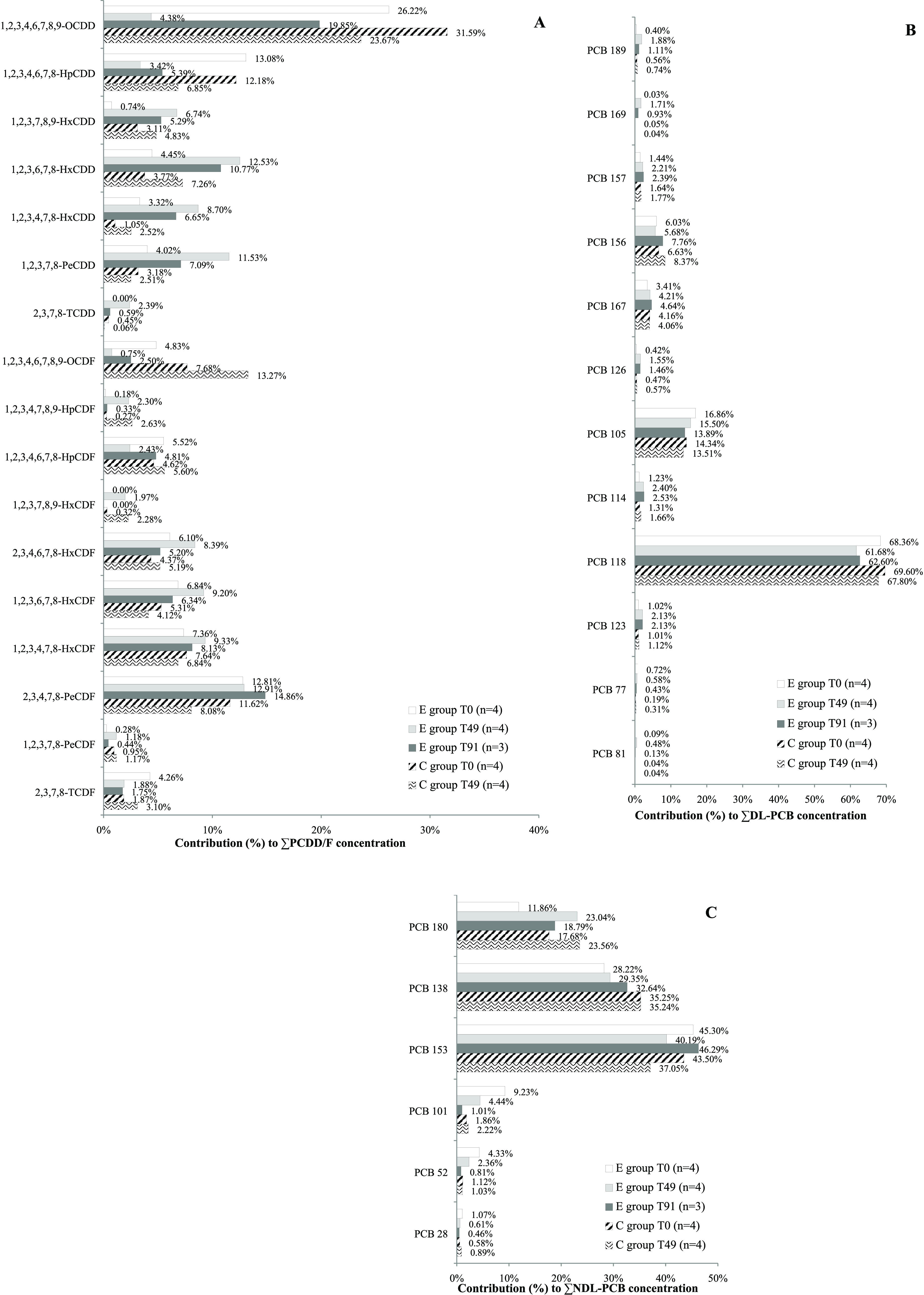
PCDD/F (A), DL-PCB (B), and NDL-PCB (C) congener patterns in milk from the experimental group (E group) at *T*_0_ (beginning of the experimental study), *T*_49_ (end of the exposure phase), and *T*_91_ (end of the monitoring of the clearance phase) and in milk from the control group (C group) at *T*_0_ and *T*_49_. The average relative contribution (%) of each congener to PCDD/F, DL-PCB, and NDL-PCB total concentrations are shown.

Concerning congener patterns (Figure 1), the most abundant PCDD/F compounds in the milk of the E group at *T*_0_ were OCDD, HpCDD, and 2,3,4,7,8-PeCDF, which accounted for 52.11% of the total PCDD/F concentration. At *T*_49_ (end of the exposure phase), PCDD/Fs in milk of E group showed a different profile: the 55.49% of PCDD/F concentration was due to 1,2,3,6,7,8-HxCDD, 2,3,4,7,8-PeCDF, PeCDD, 1,2,3,4,7,8-HxCDF, and 1,2,3,6,7,8-HxCDF. At the end of the monitoring of the clearance phase (*T*_91_), OCDD returned to characterize the milk profile of E group (Figure 1A). As shown in Figure 1A, PCDD/F profiles of C group milk at *T*_0_ and *T*_49_ were dominated by OCDD, as already seen for the E group profile at *T*_0_ and *T*_91_.

PCB 118 was the dominant DL-PCB congener in the milk samples of both E group (*T*_0_, *T*_49_, *T*_91_) and C group (*T*_0_ and *T*_49_) cows, always followed by PCBs 105, 156, and 167 (Figure 1B). The sum of these four congeners accounted for 94.65, 94.72, and 93.74% of the total DL-PCB concentration in milk of E group at *T*_0_ and in milk of C group at *T*_0_ and *T*_49_, respectively. However, their sum decreased to 87.07% in the milk of E group at *T*_49_ and 88.89% in the milk of E group at *T*_91_, due to the increase of the relative contribution of the other DL-PCB congeners, except for the less chlorinated ones (PCB 81 and PCB 77), which remained rather stable.

Regarding NDL-PCBs, the hexachlorinated congeners (PCBs 153 and 138) characterized the pattern of the milk samples of both groups (Figure 1C). These two congeners are generally the most commonly detected NDL-PCBs in raw milk and dairy products,^29^ and they were classified as the congeners found at major concentration in cow milk, together with DL-PCB 118 and NDL-PCB 180.^41^

The less chlorinated NDL-PCB compounds (PCB 28, 52, 101) were found under the respective LOQ (<1.00 ng/g fat) in all milk samples collected from the C group. PCB 28 was under the LOQ also in all milk samples obtained from E group cows. PCB 52 resulted above the LOQ only in the milk sample of cow 1E at *T*_49_ (1.21 ng/g fat). Concerning PCB 101, it was found over the LOQ only in the milk samples of E group cows at T_42_ (mean value 1.25 ng/g fat) and *T*_49_ (mean value 1.84 ng/g fat) and returned rapidly under the LOQ immediately after the end of the exposure phase. EFSA reported left censured data for NDL-PCBs 28, 52, and 101 in about 20% of the food and feed samples analyzed in Europe and these data went beyond 25% for values expressed on a fat basis.^29^ The significant metabolism of PCB 28, 52, and 101 in dairy cows, as reported by several authors,^18,21^ could explain the results obtained in the present study.

**Figure 2:**
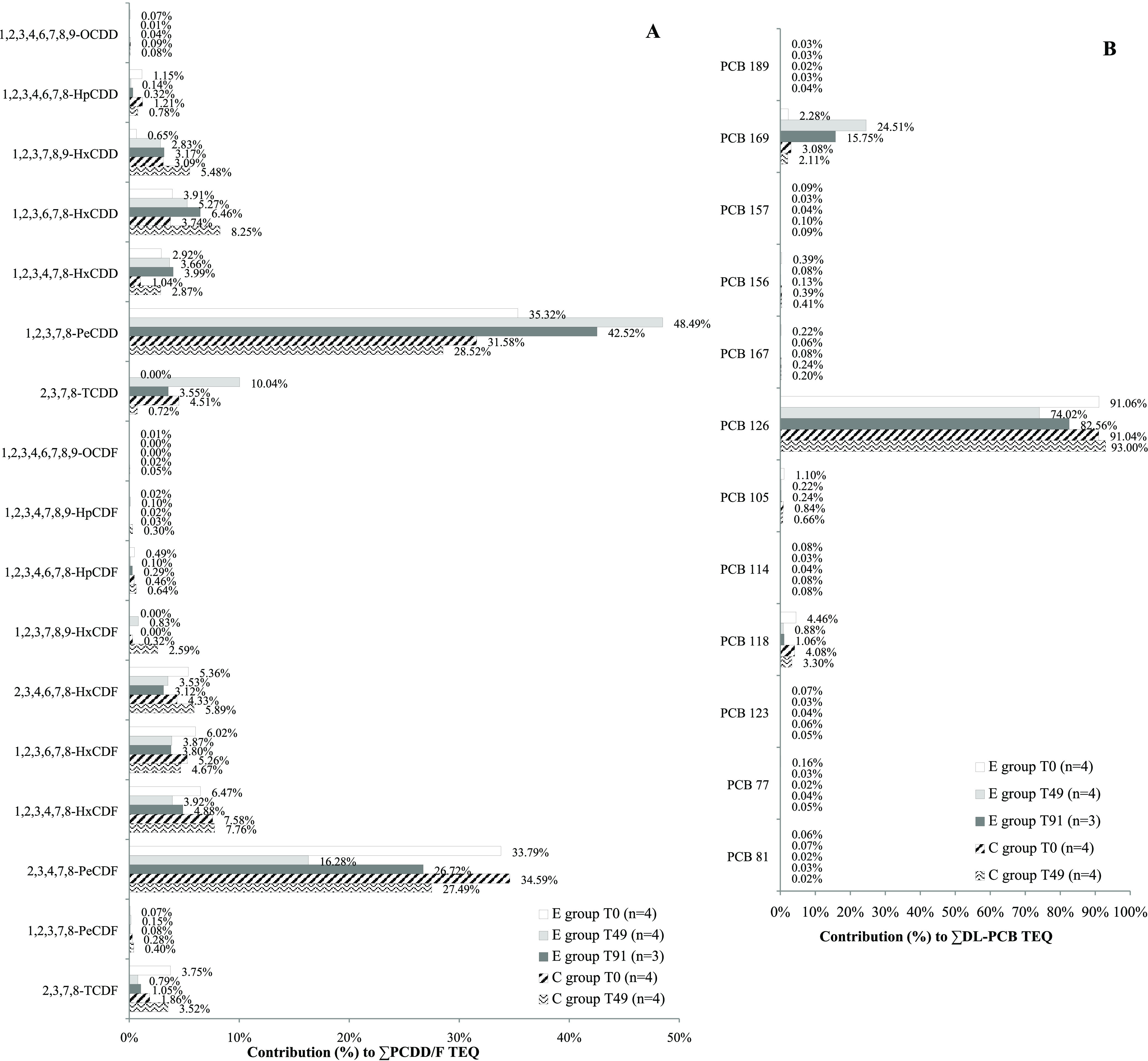
Congener average relative contribution to PCDD/F TEQ (A) and DL-PCB TEQ (B) in milk from the experimental group (E group) and from the control group (C group) at different sampling points: *T*_0_ (beginning of the experimental study), *T*_49_ (end of the E group exposure phase), and *T*_91_ (end of the monitoring of E group clearance phase).

Figure 2 shows the average relative contribution of each congener to PCDD/F TEQ (Figure 2A) and to DL-PCB TEQ (Figure 2B) in milk from E group and C group cows at different milk sampling points. In the milk collected from untreated animals (i.e., E group cows at *T*_0_; C group cows at *T*_0_ and *T*_49_), 2,3,4,7,8-PeCDF and PeCDD were the two most important congeners equally influencing the PCDD/F TEQ level (Figure 2A). In the milk from E group cows at *T*_49_ (end of the exposure phase), the relative contribution of PeCDD to the total PCDD/F TEQ increased up to 48.49%, due to the reduction of the contribution of 2,3,4,7,8-PeCDF. In the same milk samples, the relative contribution of TCDD increased up to 10.04% (it was 0.00% in the milk from E group at *T*_0_).

In the milk from E group cows at *T*_91_ (end of the monitoring of the clearance phase), PeCDD relative contribution to the total PCDD/F TEQ began to reduce, while 2,3,4,7,8-PeCDF relative contribution increased up to 26.72%. In the same milk samples, TCDD contribution decreased to 3.55%.

Concerning congener relative contribution to the total DL-PCB TEQ, PCB 126 and PCB 118 were the two most influencing compounds in the milk collected from the untreated animals (Figure 2B). At the end of the exposure phase (*T*_49_), the average relative contribution of PCB 126 to the total DL-PCB TEQ in the milk of E group cows was reduced to 74.02%, due to a substantial increase of the contribution of PCB 169. At the end of the monitoring of E group clearance phase (*T*_91_), PCB 126 relative contribution to the total DL-PCB TEQ was already 82.56%, while PCB 169 decreased to 15.75%.

### Carryover Rates

In E group cows, all 35 investigated congeners reached the SS during the exposure phase (49 days) (Table 3). Most of the PCDD/F congeners (14/17; 82.35%) reached the SS within 21 days of exposure, while the 50% of DL-PCBs (6/12) and the 66.67% of NDL-PCBs (4/6) reached the SS within 28 days of exposure (Table 3). Some authors estimated that SS in cow milk can be attained after about 3 months of continued exposure to “naturally” contaminated diets;^6,20^ however, in this study, the high contamination of the fortified corn oil, daily offered to the animals on a constant and controlled basis, could explain the obtained results as also reported in dairy goats by Costera and colleagues.^35^ Considering TEQ values, the SS was reached within 35 days both for PCDD/Fs (mean TEQ value at SS = 1.90 pg/g fat) and for DL-PCBs (mean TEQ value at SS = 7.80 pg/g fat). Huwe and Smith^24^ found that TEQ in milk reached the SS after 17 days of exposure in two cows receiving a daily dose of 135 ng TEQ.

**Table 3:** Mean Concentrations at the Steady State (SS), Days Needed To Reach the SS, and Carryover Rates (COR) of PCDD/F and PCB Congeners in Milk from the Experimental Group (*n* = 4)^a^

	this study				EFSA^6^^,^^b^	Thomas et al.^21^	Kerst et al.^47^	Diletti et al.^48^
	mean concentration at SS (pg/g fat)	achievement of SS (days)	COR	background-corrected COR^c^	COR	COR	COR	COR
	dairy cows *n* = 4	dairy cows *n* = 4	dairy cows *n* = 4	dairy cows *n* = 4	dairy cows	dairy cows *n* = 5	dairy cows *n* = 26	dairy buffaloes *n* = 3
**PCDD/Fs**								
2,3,7,8-TCDF	0.23	21	5.7	3.7	2			1.8
1,2,3,7,8-PeCDF	0.14	21	4.5	3.6	4			3.7
2,3,4,7,8-PeCDF	0.93	21	34.1	26.5	40			22.4
1,2,3,4,7,8-HxCDF	0.88	21	28.1	22.5	21			15.7
1,2,3,6,7,8-HxCDF	0.62	35	17.7	14.7	23			14.4
2,3,4,6,7,8-HxCDF	0.64	35	23.5	18.6	19			14.3
1,2,3,7,8,9-HxCDF	0.26	21	9.7	7.6	8			1.8
1,2,3,4,6,7,8-HpCDF	0.19	14	4.0	1.1	4			4.6
1,2,3,4,7,8,9-HpCDF	0.19	21	7.0	4.5	5			4.6
1,2,3,4,6,7,8,9-OCDF	0.26	21	3.8	–1.2	1			0.5
2,3,7,8-TCDD	0.23	21	43.2	42.9	35			25.9
1,2,3,7,8-PeCDD	0.84	21	31.1	28.7	33			20.4
1,2,3,4,7,8-HxCDD	0.66	21	14.8	13.3	26			12.7
1,2,3,6,7,8-HxCDD	0.79	28	29.0	22.2	34			14.8
1,2,3,7,8,9-HxCDD	0.67	21	18.3	14.9	19			7.6
1,2,3,4,6,7,8-HpCDD	0.35	21	5.6	2.8	7			5.0
1,2,3,4,6,7,8,9-OCDD	0.33	14	2.5	–2.1	1			1.8
**Non-ortho DL-PCBs**								
PCB 81	17.43	21	12.4	12.1	11		8.7	7.4
PCB 77	22.69	35	10.3	8.6	1		2.4	1.5
PCB 126	60.67	35	40.2	35.6	32		40	21.8
PCB 169	54.04	28	39.8	39.4	35		50	30.4
**Mono-ortho DL-PCB**								
PCB 123	53.98	28	37.0	27.6			8.9	27.1
PCB 118	1134.31	28	148.6	39.7		109	33	35.2
PCB 114	62.05	28	41.2	27.6			44	44.8
PCB 105	294.65	28	75.9	33.2		0	28	45.9
PCB 167	136.32	42	82.1	52.1		91	21	50.3
PCB 156	176.31	35	95.6	39.9		76	19	54.0
PCB 157	78.03	28	57.5	41.5			24	37.9
PCB 189	62.60	35	41.5	35.5			13	29.4
**NDL-PCBs**								
PCB 28	47.85	28	0.2	0.1		4		
PCB 52	415.22	42	1.4	1.3		0		
PCB 101	976.19	35	3.4	3.2		5		
PCB 153	9979.79	28	32.3	28.5		83		
PCB 138	9389.07	28	68.6	60.3		74		
PCB 180	9697.27	28	32.4	29.8		67		

a CORs obtained in other studies are also reported.

b Mean COR values based on 7 studies.^6^

c COR values corrected for the milk background of not exposed cows (control group).

PCDD/F and PCB CORs in E group were estimated using the information on feed (congener concentrations and feed intake) and on contaminant levels in milk at SS. CORs were calculated considering the contaminant intake derived from the fortified corn oil and from the background contaminations of both the TMR and the blank corn oil.

Since it was not possible to measure cows’ individual feed intake, COR values were calculated as average E group values. However, looking at an “averaged” situation may help to better understand field contamination episodes, in which only bulk milk data are generally available.^36^

Table 3 showed the estimated COR values, calculated with and without the correction for the background of the not exposed cows (C group), and it includes also data reported by other studies. For the background-corrected CORs, the contamination observed in milk from not exposed cows at *T*_49_ was subtracted, to investigate the effects of other potential sources of contamination (e.g., environment) different from feed.

Concerning PCDD/F CORs, the higher chlorinated PCDD/Fs showed, in general, lower COR values than the lower chlorinate ones, with the exceptions of TCDF and 1,2,3,7,8-PeCDF that were characterized by low CORs despite their low degree of chlorination (Table 3). As reported by other authors,^6,20,24,42,43^ high chlorinated PCDD/Fs (Cl_7_DD/F and Cl_8_DD/F) are poorly transferred to milk (≤7% in this study), due to the low absorption in the digestive tract while the weak transfers of TCDF and 1,2,3,7,8-PeCDF can be explained by their rapid hepatic degradation.^6,35,44^ PCDD/F CORs measured in this study were generally consistent with those reported in the scientific literature for dairy cows and dairy buffaloes (Table 3), and they explained very well the PCDD/F congener pattern found in E group at *T*_49_ (Figure 1A), which was characterized by congeners with high and medium CORs, while the congeners with low CORs were poorly represented.

According to McLachlan et al.,^45^ PCDD/F congeners can be divided into three groups based on COR values: (i) highly transferred congeners (TCDD, 2,3,4,7,8-PeCDF, and PeCDD); (ii) moderately transferred congeners (Cl_6_DD/F), and (iii) poorly transferred congeners (Cl_7_DD/F, Cl_8_DD/F, TCDF, and 1,2,3,7,8-PeCDF) (Table 3). In particular, the first group (high COR congeners) is made up of the 3 PCDD/Fs with the highest WHO-TEF values.^34^ This general tendency is in line with our results and with the findings of the few field studies available in the literature for dairy cows.^19,39,43,46^

PCDD/F CORs obtained in the present study were in the range of those derived from field studies.^19,39,43,46^ The predominant congeners were in line with the field findings, while the hexacongeners showed higher COR values than those found in field studies with municipal waste incinerators as sources of contamination^19,46^ but lower than those derived from field studies with citrus pulp or mineral feed supplement as contaminant sources.^39,43^ The bioavailability of PCDD/Fs from different matrices could probably have affected the values recorded in the studies.^43^

Very few papers report an estimation of PCB CORs in cow milk; in particular, it is very difficult to find data about mono-ortho DL-PCBs and NDL-PCBs.^12,44,47^ Regarding DL-PCBs, the less chlorinated congeners (PCB 81 and PCB 77) were probably rapidly metabolized or little absorbed and, as a result, they were poorly transferred to milk, as also found by other authors in several ruminant species, including dairy cows.^6,12,45,47,48^ Thus, the behavior of the less chlorinated DL-PCBs seems to matched that of TCDF rather than TCDD’s.

The other DL-PCB congeners, characterized by 5 or more chlorine atoms, showed higher COR values (>27%). Similar results were obtained by Kerst et al.,^47^ in dairy cows exposed to background levels (Table 3); by Diletti and colleagues,^48^ during a controlled feeding study in dairy buffaloes (Table 3); and by Costera and colleagues^35^ in dairy goats. However, Kerst et al.^47^ and Costera et al.^35^ found a quite low COR value for PCB 123, which was not confirmed in our study. McLachlan^18^ classified PCB 123 as a persistent congener widely excreted in cow milk, together with DL-PCB 118, 156, and 157, and our results seem to confirm this finding.

The CORs of the two DL-PCBs with the highest WHO-TEF values (i.e., PCB 126 and PCB 169) agreed well with the average values reported by EFSA^6^ and with other studies carried out in dairy cows,^12,24^ including field studies,^19,47^ and they were estimated between 35 and 40% (Table 3).

As seen for DL-PCBs, also NDL-PCBs showed a great difference in COR values between the less chlorinated congeners (PCB 28, 52, and 101) and the high chlorinated compounds (PCB 153, 138, and 180). In particular, the low chlorinated congeners showed poor transfer rates to cow milk (COR < 4%). Although these values could have been affected by the left censored data obtained in the present study; several authors reported that these congeners are largely metabolized in dairy cows^18,21^ and they are presented at minor or at moderate concentrations in cow milk.^41^ In addition, the obtained COR values agreed well with those reported by Thomas et al.^21^ (Table 3) for PCB 28, 52, and 101, who gave to the cows a background-contaminated diet in which PCB 28 was well represented. A similar behavior was also recorded in dairy goats,^35^ in which rather low COR values were found for the less chlorinated NDL-PCBs, while significantly higher CORs were obtained for the high chlorinated congeners. NDL-PCBs 153, 138, and 180 were largely transferred into milk also in the present study and in the other studies carried out in dairy cows,^18,21^ probably due to their high persistence.^18,21,35^ Papers reporting CORs of PCB 153, 138, and 180 generally showed similar values between the 3 congeners, e.g.;^18,21,35^ on the contrary, in the present study, PCB 138 showed twice the COR value of PCB 153 and 180. The lower dosage of PCB 138 in the feed (less than half the dosage of PCB 153 and PCB 180) could explain this finding; in fact, a dosage dependency of the COR had been previously reported by McLachlan.^18^

The overall COR of ∑NDL-PCBs resulted equal to 25.4% (background-corrected COR = 21.3%). The estimated CORs of ∑PCDD/F TEQ, ∑DL-PCB TEQ, and ∑PCDD/F and DL-PCB TEQ were, respectively, 27.1% (background-corrected COR = 22.9%), 40.4% (background-corrected COR = 36.5%), and 36.9% (background-corrected COR = 32.9%). Hoogenboom et al.^12^ reported similar values (18–25% for ∑PCDD/F TEQ and 32–35% for ∑DL-PCB TEQ) and underlined the strong influence on TEQ CORs of the congeners contributing most to the TEQ levels.^12^ This was confirmed also by our results: ∑PCDD/F COR TEQ was mainly affected by TCDD, 2,3,4,7,8-PeCDF and PeCDD (Figure 2A), while ∑DL-PCB COR TEQ was influenced by PCB 126 and PCB 169 (Figure 2B).

Kerst and colleagues^47^ studied the transfer of DL-PCBs and PCDD/Fs from fresh grass to cow milk at background levels and found a COR value of 36% for ∑DL-PCB TEQ, equal to the one estimated in the present study. On the other hand, they obtained a quite high COR value for ∑PCDD/F TEQ (50%), probably due to the very low levels found in grass, which resulted in higher gastrointestinal resorption rates, as stated by the authors themselves.^47^ Another field study^40^ reported a COR value of 26% for ∑PCDD/F TEQ, in agreement with our results.

### Clearance of the Contaminants

The average time needed by PCDD/Fs and PCBs to return to the basal level in milk of E group is shown in Table 4. No significant effect of cows, milk fat, and milk yield was found (*P* > 0.05). Twelve congeners (4 PCDD/Fs, 5 DL-PCBs and 3 NDL-PCBs) returned to the basal level within 7 days on clean feed, 2 within 14 days, 7 within 28 days, and 9 within 42 days (Table 4). OCDF and OCDD, which showed the lowest PCDD/F CORs, remained always very close to the basal level during the entire study period. A similar behavior was found for PCB 77, which was characterized by the lowest DL-PCB COR. On the other hand PCB 169 and PCB 153 did not return to the basal level after 42 days of monitoring of the clearance phase. Generally, the congeners showing the lower COR values returned rapidly to the basal level; in particular, this was true for PCDD/F and NDL-PCB congeners, while DL-PCBs showed a different behavior. DL-PCBs 118, 156, 167, 105, and 157, despite the relative high COR values, returned to the basal levels within 7 days on clean feed. These congeners were the most abundant congeners in milk of E cows at *T*_0_ (Figure 1B); thus, the background contamination of the cows could have affected the obtained results. In fact, these compounds were found far below the initial levels (*T*_0_) both in E group cows at *T*_91_ and in C group cows at *T*_49_.

**Table 4:** Average Time (days) Needed by PCDD/Fs and PCBs To Return to the Basal Level in the Milk of the Experimental Group (*n* = 4)

time to reach the basal level (days)			
PCDD/Fs		PCBs	
2,3,7,8-TCDF	7	**DL-PCBs**	
1,2,3,7,8-PeCDF	14	PCB 81	28
2,3,4,7,8-PeCDF	42	PCB 77	-
1,2,3,4,7,8-HxCDF	42	PCB 123	42
1,2,3,6,7,8-HxCDF	28	PCB 118	7
2,3,4,6,7,8-HxCDF	28	PCB 114	28
1,2,3,7,8,9-HxCDF	7	PCB 105	7
1,2,3,4,6,7,8-HpCDF	7	PCB 126	42
1,2,3,4,7,8,9-HpCDF	7	PCB 167	7
1,2,3,4,6,7,8,9-OCDF	-	PCB 156	7
2,3,7,8-TCDD	28	PCB 157	7
1,2,3,7,8-PeCDD	42	PCB 169	>42
1,2,3,4,7,8-HxCDD	28	PCB 189	28
1,2,3,6,7,8-HxCDD	42	∑DL-PCB TEQ	42
1,2,3,7,8,9-HxCDD	42	∑PCDD/F and DL-PCB TEQ	42
1,2,3,4,6,7,8-HpCDD	14		
1,2,3,4,6,7,8,9-OCDD	-	**NDL-PCBs**	
∑PCDD/F TEQ	42	PCB 28	7
		PCB 52	7
		PCB 101	7
		PCB 153	>42
		PCB 138	42
		PCB 180	42
		∑NDL-PCBs	>42

Concerning E group average TEQ levels, ∑PCDD/F TEQ, ∑DL-PCB TEQ, and ∑PCDD/F and DL-PCB TEQ returned to the basal level within 42 days on clean feed (Table 4).

The sum of the 6 NDL-PCB indicators did not reach the basal level during the monitoring of the clearance phase: at *T*_91_, ∑NDL-PCBs in the milk of E group was 11.49 ng/g fat (average value) while at *T*_0_, it was 8.36 ng/g fat (average value).

This study is one of the few studies dealing with the kinetics in cow milk of mono-ortho DL-PCBs and NDL-PCBs. The estimated PCDD/F and PCB COR values generally agreed well with the findings of other authors; thus, based on congeners’ behavior, PCDD/Fs and PCBs can be divided into well-defined and quite fixed groups (i.e., highly transferred, moderately transferred, and poorly transferred) and this general tendency seems to be maintained despite the different sources of contamination involved in the different studies.

Feed to milk transfer of PCDD/F and DL-PCB congeners characterized by a high WHO-TEF is fast, due to their relative high COR; as a consequence, legislative limits in cow milk can be rapidly overcome if feeding material close to EU MLs is offered to dairy cows, as also reported by Hoogenboom et al.^12^ However, the removal of the contaminated feed succeeded in restoring, after only 1 week, the compliance of cow milk showing a maximum level of contamination of 9.69 pg TEQ/g fat for the sum of PCDD/Fs and DL-PCBs and of 42.30 ng/g fat for the sum of the 6 NDL-PCBs indicators.

## Supporting Information Available

Characteristics of the dairy cows selected for the feeding study (Table S1); background contamination levels of the bulk tank milk of the selected farm (Table S2); PCDD/F and PCB content in the black corn oil and in the purchased total mixed ration (Table S3); daily average ingestion rates of the experimental and control groups (Figure S1); cow milk production (Figure S2); cow body weight (Figure S3) (PDF)

## Abbreviations Used

AL: action level
ASE: accelerated solvent extractor
bw: body weight
C: control group
COR: carryover rate
DL-PCB: dioxin-like polychlorinated biphenyl
DM: dry matter
E: experimental group
HRGC-HRMS: high-resolution gas-chromatography coupled with high-resolution mass spectrometry
LB: lower bound
LM: linear model
LOQ: limit of quantification
ML: maximum level
NDL-PCB: non-dioxin-like polychlorinated biphenyl
PCB: polychlorinated biphenyl
PCDD/F: polychlorinated dibenzo-*p*-dioxin/dibenzofuran
SS: steady state
TEQ: toxic equivalent
TMR: total mixed ration
TWI: tolerable weekly intake
UB: upper bound
WHO-TEF: World Health Organization-toxic equivalency factor

## References

[ref1] Nizzetto L.; Macleod M.; Borga K.; Cabrerizo A.; Dachs J.; Di Guardo A.; Ghirardello D.; Hansen K. M.; Jarvis A.; Lindroth A.; Ludwig B.; Monteith D.; Perlinger J. A.; Scheringer M.; Schwendenmann L.; Semple K. T.; Wick L. Y.; Zhang G.; Jones K. C. Past, present, and future controls on levels of persistent organic pollutants in the global environment. Environ. Sci. Technol. 2010, 44, 6526–6531. 10.1021/es100178f. 20604560

[ref2] Hoogenboom R.; Traag W.; Fernandes A.; Rose M. European developments following incidents with dioxins and PCBs in the food and feed chain. Food Control 2015, 50, 670–683. 10.1016/j.foodcont.2014.10.010.

[ref3] Weber R. Learning from dioxin & PCBs in meat—problems ahead?. IOP Conf. Ser. Earth Environ. 2017, 85, 012002 10.1088/1755-1315/85/1/012002.

[ref4] Weber R.; Herold C.; Hollert H.; Kamphues J.; Ungemach L.; Blepp M.; Ballschmiter K. Life cycle of PCBs and contamination of the environment and of food products from animal origin. Environ. Sci. Pollut. Res. 2018, 25, 16325–16343. 10.1007/s11356-018-1811-y. 29589245

[ref5] Weber R.; Herold C.; Hollert H.; Kamphues J.; Blepp M.; Ballschmiter K. Reviewing the relevance of dioxin and PCB sources for food from animal origin and the need for their inventory, control and management. Environ. Sci. Eur. 2018, 30, 42 10.1186/s12302-018-0166-9. 30464877 PMC6224007

[ref6] Risk for animal and human health related to the presence of dioxins and dioxin-like PCBs in feed and food. EFSA J. 2018, 16, 5333 10.2903/j.efsa.2018.5333. PMC7009407 32625737

[ref7] No 1259/2011 of 2 December 2011 amending Regulation (EC) No 1881/2006 as regards maximum levels for dioxins, dioxin-like PCBs and non dioxin-like PCBs in foodstuffs. Off. J. Eur. Union 2011, L 320, 18–23.

[ref8] No 277/2012 of 28 March 2012 amending Annexes I and II to Directive 2002/32/EC of the European Parliament and of the Council as regards maximum levels and action thresholds for dioxins and polychlorinated biphenyls. Off. J. Eur. Union 2012, L 91, 1–7.

[ref9] Commission Recommendation of 3 December 2013 on the reduction of the presence of dioxins, furans and PCBs in feed and food (2013/711/EU). Off. J. Eur. Union 2013, L 323, 37–39.

[ref10] Malisch R.; Kotz A. Dioxins and PCBs in feed and food – Review from European perspective. Sci. Total Environ. 2014, 491–492, 2–10. 10.1016/j.scitotenv.2014.03.022. 24804623

[ref11] Hoogenboom L. A.; Kan C. A.; Zeilmaker M. J.; Van Eijkeren J.; Traag W. A. Carry-over of dioxins and PCBs from feed and soil to eggs at low contamination levels – influence of mycotoxin binders on the carry-over from feed to eggs. Food Addit. Contam. 2006, 23, 518–527. 10.1080/02652030500512037. 16644600

[ref12] Hoogenboom R. L. A. P.; Klop A.; Herbes R.; van Eijkeren J. C. H.; Zeilmaker M. J.; van Vuuren A. M.; Traag W. A. Carry-over of polychlorinated dibenzo-p-dioxins and dibenzofurans (PCDD/Fs) and polychlorinated biphenyls (PCBs) in dairy cows fed smoke contaminated maize silage or sugar beet pulp. Chemosphere 2015, 137, 214–220. 10.1016/j.chemosphere.2015.07.040. 26253955

[ref13] Piskorska-Pliszczynska J.; Maszewski S.; Mikolajczyk S.; Pajurek M.; Strucinski P.; Olszowy M. Elimination of dioxins in milk by dairy cows after the long-term intake of contaminated sugar beet pellets. Food Addit. Contam., Part A 2017, 34, 842–852. 10.1080/19440049.2017.1300943. 28402200

[ref14] Diletti G.; Ceci R.; Scortichini G.; Migliorati G. Dioxin levels in livestock and grassland near a large industrial area in Taranto (Italy). Organohalogen Compd. 2009, 71, 002344.

[ref15] Esposito M.; Cavallo S.; Serpe F. P.; D’Ambrosio R.; Gallo P.; Colarusso G.; Pellicano R.; Baldi L.; Guarino A.; Serpe L. Levels and congener profiles of polychlorinated dibenzo-p-dioxins, polychlorinated dibenzofurans and dioxin-like polychlorinated biphenyls in cow’s milk collected in Campania, Italy. Chemosphere 2009, 77, 1212–1216. 10.1016/j.chemosphere.2009.09.011. 19836049

[ref16] Turrio-Baldassarri L.; Alivernini S.; Carasi S.; Casella M.; Fuselli S.; Iacovella N.; Iamiceli A. L.; La Rocca C.; Scarcella C.; Battistelli C. L. PCB, PCDD and PCDF contamination of food of animal origin as the effect of soil pollution and the cause of human exposure in Brescia. Chemosphere 2009, 76, 278–285. 10.1016/j.chemosphere.2009.03.002. 19345979

[ref17] Jensen D. J.; Hummel R. A. Secretion of TCDD in milk and cream following the feeding of TCDD to lactating dairy cows. Bull. Environ. Contam. Toxicol. 1982, 29, 440–446. 10.1007/BF01605609. 6890865

[ref18] McLachlan M. S. Mass balance of polychlorinated biphenyls and other organochlorine compounds in a lactating cow. J. Agric. Food Chem. 1993, 41, 474–480. 10.1021/jf00027a024.

[ref19] Slob W.; Olling M.; Derks H. J. G. M.; de Jong A. P. J. M. Congener-specific bioavailability of PCDD/Fs and coplanar PCBs in cows: Laboratory and field measurements. Chemosphere 1995, 31, 3827–3838. 10.1016/0045-6535(95)00256-8. 7583023

[ref20] McLachlan M.; Richter W. Uptake and transfer of PCDD/Fs by cattle fed naturally contaminated feedstuffs and feed contaminated as a result of sewage sludge application. 1. Lactating cows. J. Agric. Food Chem. 1998, 46, 1166–1172. 10.1021/jf970922u. 11743775

[ref21] Thomas G. O.; Sweetman A. J.; Jones K. C. Input–output balance of polychlorinated biphenyls in a long-term study of lactating dairy cows. Environ. Sci. Technol. 1999, 33, 104–112. 10.1021/es980322r.

[ref22] Lorenzi V.; Ghidini S.; Angelone B.; Ferretti E.; Menotta S.; Fedrizzi G.; Varisco G.; Foschini S.; Diegoli G.; Bertocchi L. Three years of monitoring of PCDD/F, DL-PCB and NDL-PCB residues in bovine milk from Lombardy and Emilia Romagna regions (Italy): Contamination levels and human exposure assessment. Food Control 2016, 68, 45–54. 10.1016/j.foodcont.2016.03.034.

[ref23] Bertocchi L.; Ghidini S.; Fedrizzi G.; Lorenzi V. Case-study and risk management of dioxins and PCBs bovine milk contaminations in a high industrialized area in Northern Italy. Environ. Sci. Pollut. Res. 2015, 22, 9775–9785. 10.1007/s11356-015-4146-y. 25637240

[ref24] Huwe J. K.; Smith D. J. Laboratory and on-farm studies on the bioaccumulation and elimination of dioxins from a contaminated mineral supplement fed to dairy cows. J. Agric. Food Chem. 2005, 53, 2362–2370. 10.1021/jf0480997. 15769182

[ref25] United States Environmental Protection Agency Office of Water Engineering and Analysis Division. Tetra-through octa-chlorinated dioxins and furans by isotope dilution HRGC/HRMS: Method 1613 Revision B October 1994. US EPA Method 1613/B, 1994.

[ref26] United States Environmental Protection Agency Office of Water Engineering and Analysis Division. Chlorinated biphenyl congeners in water, soil, sediment, biosolids, and tissue by HRGC/HRMS: Method 1668 Revision C April 2010. US EPA Method 1668/C, 2010.

[ref27] European Commission Preparatory Actions in the Field of Dioxin and PCBs; European POPs Expert Team: Brussels, Belgium, 2002.

[ref28] Turrio-Baldassarri L.; Abate V.; Alivernini S.; Battistelli C. L.; Carasi S.; Casella M.; Iacovella N.; Iamiceli A. L.; Indelicato A.; Scarcella C.; La Rocca C. A study on PCB, PCDD/PCDF industrial contamination in a mixed urban-agricultural area significantly affecting the food chain and the human exposure. Part I: Soil and feed. Chemosphere 2007, 67, 1822–1830. 10.1016/j.chemosphere.2006.05.124. 17234238

[ref29] Results of the monitoring of non dioxin-like PCBs in food and feed. EFSA J. 2010, 8, 1701 10.2903/j.efsa.2010.1701. PMC13085141 42005761

[ref30] No 152/2009 of 27 January 2009 laying down the methods of sampling and analysis for the official control of feed. Off. J. Eur. Union 2009, L 54, 1–130.

[ref31] No 691/2013 of 19 July 2013 amending Regulation (EC) No 152/2009 as regards methods of sampling and analysis. Off. J. Eur. Union 2013, L 197, 1–12.

[ref32] No 252/2012 of 21 March 2012 laying down methods of sampling and analysis for the official control of levels of dioxins, dioxin-like PCBs and non-dioxin-like PCBs in certain foodstuffs and repealing Regulation (EC) No 1883/2006. Off. J. Eur. Union 2012, L 84, 1–22.

[ref33] No 1069/2009 of the European Parliament and of the Council of 21 October 2009 laying down health rules as regards animal by-products and derived products not intended for human consumption and repealing Regulation (EC) No 1774/2002 (animal by-products regulation). Off. J. Eur. Union 2009, L 300, 1–33.

[ref34] Van den Berg M.; Birnbaum L. S.; Denison M.; De Vito M.; Farland W.; Feeley M.; Fiedler H.; Hakansson H.; Hanberg A.; Haws L.; Rose M.; Safe S.; Schrenk D.; Tohyama C.; Tritscher A.; Tuomisto J.; Tysklind M.; Walker N.; Peterson R. E. The 2005 World Health Organization reevaluation of human and mammalian toxic equivalency factors for dioxins and dioxin-like compounds. Toxicol. Sci. 2006, 93, 223–241. 10.1093/toxsci/kfl055. 16829543 PMC2290740

[ref35] Costera A.; Feidt C.; Marchand P.; Le Bizec B.; Rychen G. PCDD/F and PCB transfer to milk in goats exposed to a long-term intake of contaminated hay. Chemosphere 2006, 64, 650–657. 10.1016/j.chemosphere.2005.10.052. 16337990

[ref36] Sweetman A. J.; Thomas G. O.; Jones K. C. Modelling the fate and behaviour of lipophilic organic contaminants in lactating dairy cows. Environ. Pollut. 1999, 104, 261–270. 10.1016/S0269-7491(98)00177-8.

[ref37] Core Team R. R: A Language and Environment for Statistical Computing; R Foundation for Statistical Computing: Vienna, Austria, 2014. http://www.R-project.org/.

[ref38] Fries G. F. Polychlorinated biphenyl residues in milk of environmentally and experimentally contaminated cows. Environ. Health Perspect. 1972, 1, 55–59. 10.1289/ehp.720155. 17539087 PMC1474869

[ref39] Brambilla G.; Fochi I.; Falce M.; De Filippis S. P.; Ubaldi A.; di Domenico A. PCDD and PCDF depletion in milk from dairy cows according to the herd metabolic scenario. Chemosphere 2008, 73, S216–S219. 10.1016/j.chemosphere.2007.11.071. 18462776

[ref40] Hoogenboom R.; Zeilmaker M.; van Eijkeren J.; Kan K.; Mengelers M.; Luykx D.; Traag W. Kaolinic clay derived PCDD/Fs in the feed chain from a sorting process for potatoes. Chemosphere 2010, 78, 99–105. 10.1016/j.chemosphere.2009.10.016. 19889443

[ref41] Sewart A.; Jones K. C. A survey of PCB congeners in U.K. cows’ milk. Chemosphere 1996, 32, 2481–2492. 10.1016/0045-6535(96)00141-5. 8653383

[ref42] Fries G. F.; Paustenbach D. J.; Luksemburg W. J. Complete mass balance of dietary polychlorinated dibenzo-p-dioxins and dibenzofurans in dairy cattle and characterization of the apparent synthesis of hepta- and octachlorodioxins. J. Agric. Food Chem. 2002, 50, 4226–4231. 10.1021/jf020037y. 12105950

[ref43] Malisch R. Increase of the PCDD/F-contamination of milk, butter and meat samples by use of contaminated citrus pulp. Chemosphere 2000, 40, 1041–1053. 10.1016/S0045-6535(99)00352-5. 10739045

[ref44] Rychen G.; Jurjanz S.; Toussaint H.; Feidt C. Dairy ruminant exposure to persistent organic pollutants and excretion to milk. Animal 2008, 2, 312–323. 10.1017/S1751731107001139. 22445026

[ref45] McLachlan M. S.; Thoma H.; Reissinger M.; Hutzinger O. PCDD/F in an agricultural food chain - Part 1: PCDD/F mass balance of a lactating cow. Chemosphere 1990, 20, 1013–1020. 10.1016/0045-6535(90)90214-E.

[ref46] Schuler F.; Schmid P.; Schlatter C. Transfer of airborne polychlorinated dibenzo-p-dioxins and dibenzofurans into dairy milk. J. Agric. Food Chem. 1997, 45, 4162–4167. 10.1021/jf970248g.

[ref47] Kerst M.; Waller U.; Reifenhäuser W.; Körner W. Carry-over rates of dioxin-like PCB from grass to cows’ milk. Organohalogen Compd. 2004, 66, 2412–2415.

[ref48] Diletti G.; Ceci R.; Ippoliti C.; Ferri N.; Marchi E.; Pirito L.; Scortichini G. PCDD/F and DL-PCB transfer to milk in buffaloes exposed to contaminated feed. Organohalogen Compd. 2014, 76, 1557–1560.

