# Supplementary material for: PCDD/Fs, DL-PCBs, and NDL-PCBs in Dairy Cows: Carryover in Milk from a Controlled Feeding Study
Source: J Agric Food Chem. 2020 Feb 5;68(7):2201–13. doi: 10.1021/acs.jafc.9b08180 (PMC7997377; doi:10.1021/acs.jafc.9b08180)
Supplement: Supplementary file 1 — jf9b08180_si_001.pdf [file jf9b08180_si_001.pdf]

SUPPORTING INFORMATION

PCDD/Fs, DL-PCBs and NDL-PCBs in dairy cows: carry-over in milk from a controlled feeding study

Valentina Lorenzi[a], Barbara Angelone[a], Enrica Ferretti[a], Andrea Galli[b], Mauro Tonoli[a], Matteo Donati[a], Francesca Fusi[a], Giorgio Zanardi[a], Sergio Ghidini[c,*], Luigi Bertocchi[a]

[a]Istituto Zooprofilattico Sperimentale della Lombardia e dell'Emilia Romagna "Bruno Ubertini", Via Antonio Bianchi 9, 25124 Brescia, Italy

[b]CREA, Research Centre for Animal production and Aquaculture, via Antonio Lombardo 11, 26900 Lodi, Italy

[c]Department of Food Science, Parma University, Via del Taglio 10, 43126 Parma, Italy

*Corresponding author at: Department of Food Science, Parma University, Via del Taglio 10, 43126 Parma, Italy. E-mail address: sergio.ghidini@unipr.it (S. Ghidini), tel. 0521902761, fax 0521902752

Table S1. Characteristics of the 8 dairy cows selected for the feeding study.

Subject ID	Date of birth	Day in milk at T_0	Lactation number	Milk yield at T_0[a]	Body weight at T_0[a]
**Experimental group (E)**					
1E	14.10.2010	119	2	41 kg/day	632 kg
2E	05.03.2011	6	2	28 kg/day	700 kg
3E	24.08.2011	110	1	24 kg/day	566 kg
4E	13.10.2011	12	1	19 kg/day	586 kg
**Control group (C)**					
1C	24.06.2010	129	2	24 kg/day	558 kg
2C	08.03.2011	8	2	31 kg/day	640 kg
3C	14.07.2011	112	1	24 kg/day	516 kg
4C	26.09.2011	28	1	25 kg/day	614 kg

[a]T_0 = Time zero (beginning of the experimental study)

Table S2. Background contamination levels of the bulk tank milk of the farm in which the study was carried out. Bulk tank milk was tested before starting the experiment to ensure that the farm was under the European maximum (MLs) and action levels (ALs) set for dioxins (PCDD/Fs), dioxin-like PCBs (DL-PCBs) and non-dioxin-like PCBs (NDL-PCBs)[1,2]. Values are reported as upperbound (lowerbound in square brackets).

	Bulk tank milk	European MLs	European ALs
ΣPCDD/Fs (pg TEQ/g fat)	0.20 [0.07]	2.50	1.75
ΣDL-PCBs (pg TEQ/g fat)	0.88 [0.85]	-	2.00
ΣPCDD/Fs and DL-PCBs (pg TEQ/g fat)	1.09 [0.91]	5.50	-
ΣNDL-PCBs[a] (ng/g fat)	7.22 [3.22]	40	-

[a]Sum of the six NDL-PCB indicators (PCB 28, 52, 101, 138, 153 and 180)

Table S3. PCDD/F and PCB levels in the blank corn oil and in the total mixed ration (TMR). Data are reported as upperbound (lowerbound in square brackets) and expressed on 12% moisture content.

	Corn oil (blank)	TMR
ΣPCDD/Fs (ng TEQ/kg)	0.17 [0.00]	0.18 [0.02]
ΣDL-PCBs (ng TEQ/kg)	0.14 [0.00]	0.14 [0.00]
ΣPCDD/Fs and DL-PCBs (ng TEQ/kg)	0.32 [0.00]	0.33 [0.02]
ΣNDL-PCBs[a] μg/kg	3.00 [0.00]	3.00 [0.00]

[a]Sum of the six NDL-PCB indicators (PCB 28, 52, 101, 138, 153 and 180)

Figure S1. Daily average ingestion rates of experimental and control groups during exposure (day 1 - 49) and clearance phases (day 50 - 91). Cow 1E, belonging to the experimental group, was excluded from the study at day 67 due to health problems.

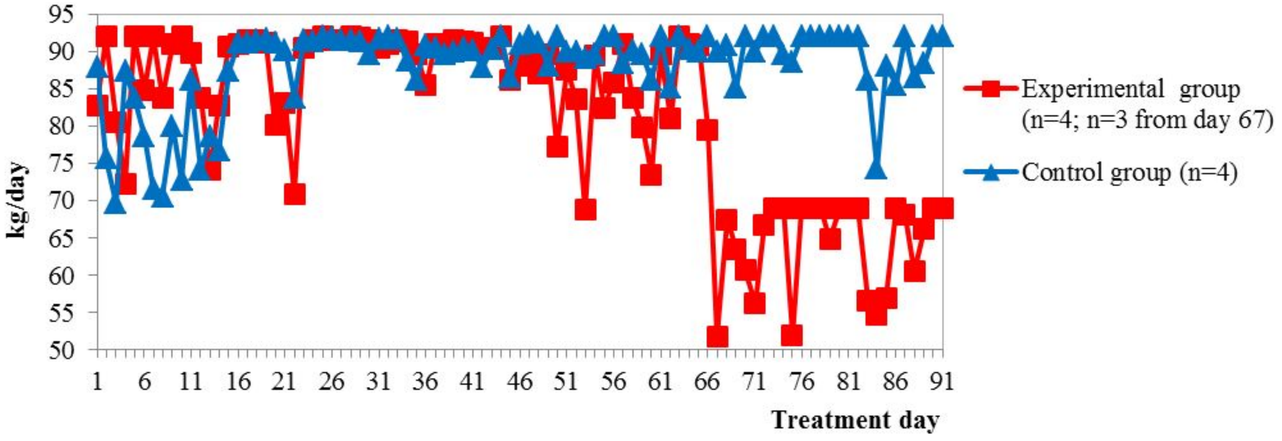

Figure S2. Production of milk by cows of the experimental (A) and control groups (B) at T_0 (beginning of the experimental study) and during the exposure (days 1 - 49) and the clearance phases (days 50 - 91). Cow 1E was excluded from the study at day 67 due to health problems.

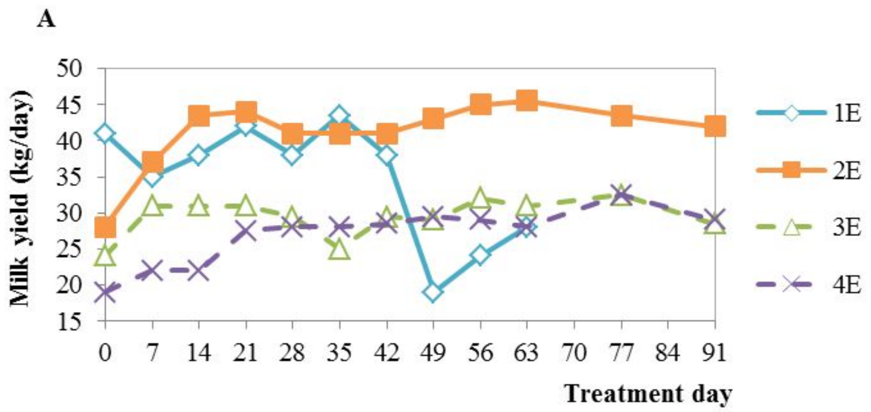

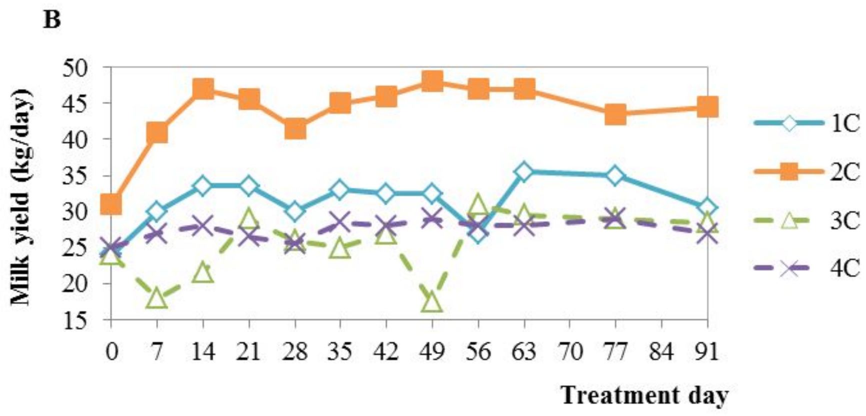

Figure S3. Body weight of cows of the experimental (A) and control groups (B) at T_0 (beginning of the experimental study) and during the exposure (days 1 - 49) and the clearance phases (days 50 - 91). Cow 1E was excluded from the study at day 67 due to health problems.

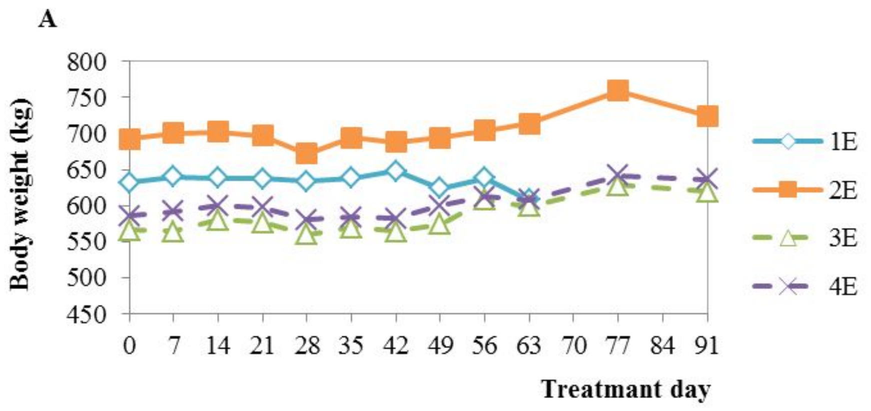

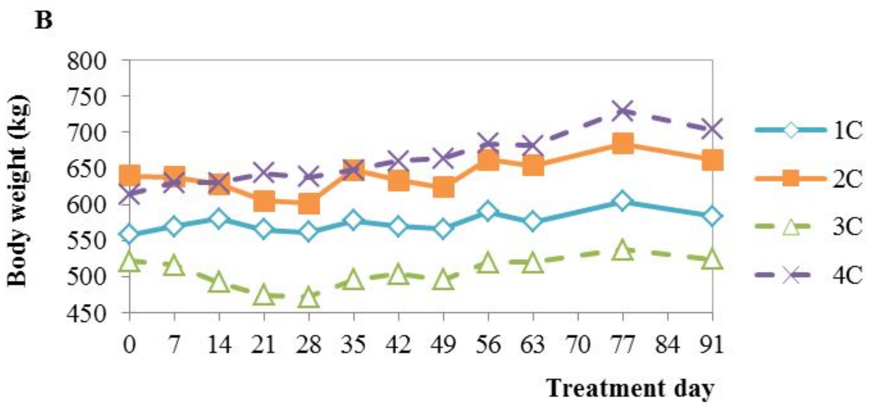

REFERENCES

(1) Commission Regulation (EU) No 1259/2011 of 2 December 2011 amending Regulation (EC) No 1881/2006 as regards maximum levels for dioxins, dioxin-like PCBs and non dioxin-like PCBs in foodstuffs. Off. J. Eur. Union 2011, L 320, 18-23.

(2) Commission Recommendation of 3 December 2013 on the reduction of the presence of dioxins, furans and PCBs in feed and food (2013/711/EU). Off. J. Eur. Union 2013, L 323, 37-39.
